# Framing Value Based Healthcare in Practice: Introducing the Complex Case and Recovery Management Framework (The CCaRM)

**DOI:** 10.5334/ijic.5975

**Published:** 2023-01-09

**Authors:** Mark Spurrell, Lorraine Potts, Amy Shaw

**Affiliations:** 1 Alliance Manchester Business School, University of Manchester and Niche Consulting, UK; 2 Your Care Strategy Ltd., UK; 3 Blackpool Teaching Hospitals NHS Foundation Trust, UK; 4 Mersey Care NHS Trust, UK

**Keywords:** value in healthcare, care platforms, case level service integration

## Abstract

**Introduction:**

There is a gap between aspiring to co-produce and co-create value in integrated healthcare and realising that in practice, particularly with complex needs and multiple stakeholders. Key principles from literature on value-based healthcare offer a conceptual framework for building suitable care platforms to support practice. This paper outlines the Complex Care and Recovery Management Framework (CCaRM) as an example of co-platforming value-based healthcare within case level practice.

**Description:**

The CCaRM was co-produced with clinicians and service users in a learning disability service. Highlighted are 6 value-making themes for building collaborative value over time, alongside case management. “Experience-in-use” was that it made sense to participants, and activated service-users and clinicians. Further empirical evaluation is needed.

**Discussion:**

There was encouragement that the CCaRM approach was implementable. Alongside further evaluative work, key issues would be: collaborating with local participants; supporting training; reconciling case-level perspectives with wider systems. Progressing integrated value-based healthcare involves: refreshing focus on the case-based view; ways of operationalising complexity; value-based case management; customisation of care styles and “democratic outcomes” within co-platforming systems.

**Conclusion:**

In principle, the CCaRM contributes to operationalising collaborative value-based healthcare for complex cases. It surfaces further research themes to refocus value and integrated care thinking. Further empirical work is needed.

## Introduction

In integrated healthcare, there is increasing emphasis on how value is generated within individual cases [1,2,3]. However, suitable frameworks are not available for complex case-level practice [4]. The many reported care programmes in the literature form a heterogenous group whose effectiveness and conceptual underpinnings are unclear [5]. Thus, better supporting complex case management remains an important contemporary issue [5,6,7,8]. A helpful response is to pragmatically adopt a value-based stance, operationalising complexity within case-level practice. Such transformation is particularly pertinent to inherently complex services, such as for Intellectual Disability and Autism (ID&A) [9]. The Complex Case and Recovery Management Framework (The CCaRM), as introduced in this paper, aims to address that gap. It combines a value-based healthcare stance with a concept of co-platforming service to address the needs of complex ID&A care at the case level. This concept has relevance for framing value-based healthcare in practice more widely. A case study format is ideally suited for developing practice and theory support for complex areas of practice [10]. A UK specialist ID&A in-patient service, within a Mental Health Trust, provided a suitable setting for exploring complex case management. This paper provides a pragmatic case study exploration of the co-development and experience in use of the CCaRM within such a setting.

As a pragmatic inquiry [11], the methodology involves outlining the pertinent conceptual background of value-based healthcare (VBHC) and “The Complex Case”. Next, is the co-development of the CCaRM approach, followed by the highlight report of a pilot evaluation of the CCaRM in use, based on reflections from workshops, service-user and practitioner surveys and an evaluation diary. Lastly, by developing the interplay between conceptualisation and experience, inferences for wider practice and theory are explored. In this context, the developing concept of co-platforming care is positioned as a feasible basis for progressing and researching VBHC within integrated care.

## Background

### Value-Based Healthcare

Value-based healthcare (VBHC) links with work by Porter and colleagues [12,13,14]. The core idea is that organising care around value generation should drive services [12]. It is a prominent theme for the integrated care agenda [3]. In this context, “Value” is that, following a service, service-users are better off than before [12,14]. Recent literature frames value as being “co-created” within such service exchanges. Detailed elsewhere, there is an intricate literature on the value co-creation concept in healthcare [1,2,15,16]. The heart of the matter is a logic shift away from providing benefit to service-users as consumers. Rather, service-users create benefit for themselves, which service providers facilitate [14]. Whilst more refinement is needed about co-creation in healthcare [14,17,18], it importantly shifts perspective from working *for* people to collaborating *with* people [19,20]. The challenge for realising VBHC is translating such collaborative value realisation into service platforms that can cope with the complex realities of healthcare in practice [21,22]. Care platforms, or service platforms, are systems for structuring and mediating relationships between participants and value generating processes for the particular case [23]. Practitioners may have developed varied eclectic arrangements to suit local customs, however a more systematic and collaborative structural underpinning is needed to serve the aim of VBHC. We adopt the term “co-platforming” to capture this shift.

### The Complex Case

Defining the “complex case” is problematic in healthcare. Prominent approaches are condition-focussed, with descriptive qualifications. For example, Wagner’s chronic care model (the CCM) focuses on “long term conditions” [24,25]; NICE guidelines use “multi-morbidity” as betokening complexity [26]. Further additional influences, such as psychosocial factors, might also be cited. However, with this approach, there are always more factors that could have been considered. These might include capacity of service-users to participate, competing priorities from multiple stakeholders, and system factors for example [27]. For a case, an alternative VBHC stance is, we argue, that “complexity” involves the broad mapping of all the moving parts to be considered so as to collaboratively generate value. Within the spirit of “co-platforming”, as we have termed it, it is something discovered *with* people, rather than ascribed *to* people. In this context, the complex case forms a service entity in its own right [1]: an integrated care project focusing on value generation, where promises can be made and kept [28]. This involves an expectation of “sense-making”, whereby participants seek to understand and reconcile ambiguous, equivocal or confusing issues or events [29]. A more detailed outline of “sense-making” is beyond the scope of this paper, but it is implicit in many case management approaches, for example the Care Programme Approach (CPA) in UK mental healthcare, [7,30,31]. This paper explores a case example of how such a framing of complex case management might be achieved in practice.

## Method

A case study format is ideally suited for developing practice and theory support for complex areas of practice [10]. A UK specialist ID&A in-patient service, within a Mental Health Trust, was chosen as a case example. Service-users typically had multiple health and social needs. Many had significant risk profiles, or difficulties with capacity and engagement. There were further legal and service policy considerations to be accommodated, as might be expected. Thus, all cases represented significant complex care challenges, however that might be defined. As part of wider efforts towards transforming the experience of care for these service-users [9], there was an aim to develop a value-based care framework, which became the CCaRM. The role of the authors was to develop the conceptual groundwork and to facilitate the co-production and piloting of the CCaRM approach, for which ethical approval was not required. The findings comprise the report of the development of the CCaRM approach and highlights of experience in use. All personal information has been kept confidential.

## Developing the CCaRM Approach

As stated, the CCaRM approach was developed as a co-production with clinicians, service-users, and service managers within a specialist ID&A service. Drawing on VBHC thinking, a series of mapping workshops were held with teams across the service. Their focus was the mapping of practices against “what might generate value” for a series of particular cases of concern within the service. Consistent with VBHC literature [12], the 6 core themes that emerged were (in easy-read wording):

– Having a helpful network of support
– Developing a shared understanding of need
– Making progress with problem areas
– Making progress with social functioning
– Avoiding harm
– Generating momentum along the care pathway

**Figure 1:**
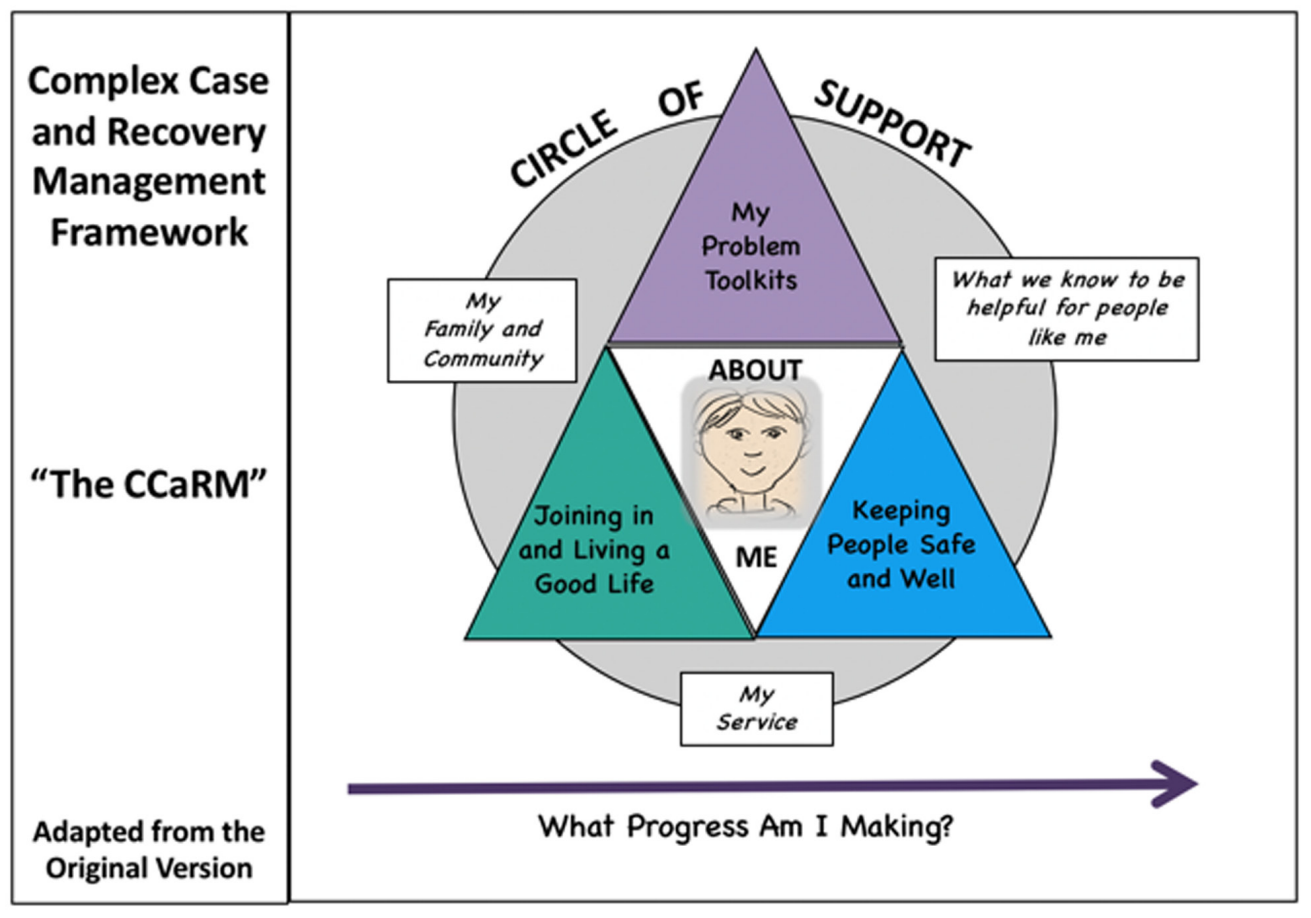
The Complex Case and Recovery Management Framework (The CCaRM) in user friendly language [32]: *Adapted and reproduced with permission of the authors*.

A first framework version was drafted. Then, a series of repeat mapping exercises were conducted with teams, leading to further revisions. Service-user workshops were conducted to explore the salience of the drafted framework for them, and to ensure that representations and language were user-friendly. The resulting easy-read CCaRM framework as a case level platform for capturing value themes is illustrated below (Figure 1). This formed the basis for the CCaRM approach.

### The CCaRM Process

In essence, the CCaRM approach is enacted as a golden thread of collaborative conversations, structured by the 6 core value themes (see Figure 1), outlined in further detail in Appendix 1. Collaboration commences with conversations between service-users and key workers. These are contextualised within broader multi-disciplinary team discussions, perhaps involving some other key stakeholders (eg family members or advocates). These are further contextualised over time within regular, multi-stakeholder, case management reviews. The focus is on the individual and their particular presenting predicament. The 6 core value themes are.

#### The Circle of Support (My Family and Community/My services/What we know to be helpful)

Context is crucial for value in healthcare [33]. Thus, it is important to understand each care project as involving a particular service user, and a unique service delivery network of supporters [33,34], or “*Circle of Support*”. The first theme, therefore, explores circle-of-support building, cutting across service systems to pull in the right people for the case as needs be [33]. In practice, this might include combinations of service user, family, clinicians, commissioners and relevant others, with all their useful knowledge and skill. It involves discussing with the service user how well it might be working already, and any concerns about that. Then, in further discussion, who further might be helpful to involve, or could existing supporters be configured better?

#### About Me

The second theme, “*About Me*”, explores ways of engaging and developing a shared understanding. In other words, what view does the service-user take of their difficulties, and to what extent is everyone in tune with that? The process would be to discuss similarities and differences in views between service-user, family, professionals etc, and what fresh initiatives might help reach a more integrated understanding: this might include collating information, making fresh assessments, and developing a shared formulation. This work might include assessments of communication needs, and education needs for the service user, and indeed other participants: service-users and family often have much to teach professionals.

#### My Problem Toolkits

The third, “*My Problem Toolkits*”, is about defining distinct problem areas for attention. The process would be to pragmatically agree a set of headings that make best sense both to the service-user and others, and which might usefully inform the development of collaborative valued care plans. It is helpful to pin down how many distinct problem areas are needing attention. In the field of ID&A, for example, it is important to shift from an unstructured focus on “challenging behaviour” to a more structured underpinning of what might be behind that, and how to address that. From such a platforming flows the exploring of progress already made with each, and the development of a suitable toolkit for further assessment and collaborative care management.

#### Joining in and Living a Good Life

Meanwhile, “*Joining in and Living a Good Life*”, explores aspects of social skills, social function and recovery. It is a key aspect of the CCaRM approach that tools and concepts are pulled into play as they might be useful. Here, for example, the “Good Life” signposts the usefulness of “the Good Lives Model” [35], a widely used tool to help structure meaning with people in services. There are many other models from the recovery toolbox that can also be considered here. Again, collaborative care planning flows from the resultant insights, looking at what is working already, and what further assessments and initiatives to pursue.

#### Keeping People Safe and Well

The fifth theme, “*Keeping People Safe and Well*”, sets the stage for exploring and mitigating areas that ought to be of concern. This would involve exploring with the service-user their understanding of risks, what mitigation might be working and what further support would be helpful. Again, contemporary tools or guidelines can be available to support this work. Also, other areas where harm might arise would need consideration. Thus, discussion would include suitability of medication, consideration of wider physical and mental wellbeing, appropriate approaches to supporting capacity and promoting autonomy. A particularly important issue within this theme, certainly within ID&A practice, is the mitigation of stigma and the managing of service-user reputation and being able to make a fresh start.

#### What Progress Am I Making?

The sixth theme captures the making of progress with “what matters” (i.e. “value”). There are two aspects to this. First, “what matters” is discovered and shaped by all the participants within the particular case. In other words, for each of the 6 value themes within the framework, value is realised in conversation by shared reflection and judgement on where progress is being made, where it is not, and what fresh ideas might be available. Further, it is important that, in addition to service-user benefits, all participants also create value for themselves within these themes [2,14,18,36]. In this context, formal CPA case management reviews are crucial touchpoints for such collaborative value-making. Thus, the second aspect to making progress is the performance of valuations as part of the case management process. Within the CCaRM process, CPA reviews are structured to support the generation of “democratic outcomes” as a collaborative perspective on progress. The format is for CPA reviews to conclude by asking service users and all other attendees to vote for each value theme on whether progress had been made, was unchanged, or slipped back since the previous review. Such categorical “win/lose” styles of outcome feature prominently in service-system thinking [37]. The vote is further enhanced by a rich discussion as to what might therefore need to happen further, moving from case review to case review. A “democratic outcome” perspective on progress would complement the deployment of more standardised outcome measures. Such co-valuations are important for reconciling different ideas about value within care projects, and are of increasing interest for healthcare practice [38].

### The CCaRM in Use

As part of the co-production of the CCaRM approach within the service, a six-month pilot evaluation was undertaken. Three units were evaluated prospectively, pre- and post-implementation of the CCaRM approach. A mixed qualitative approach resulted in data collection from several sources, including service-user and staff surveys, workshops, routine incident reporting and thematic analysis of a research diary. The research diary was completed by AS, whilst acting as project lead for the implementation pilot. With turnover of patients and staff, and fluctuations in presentation, responses were pooled from across the units. Reported here is an overview of findings, developed to serve as an illustration of “experience-in-use” of the CCaRM. The full pilot report was published internally, informing further roll-out of co-platforming practice. Participants consented to anonymised responses being featured in wider publication.

#### Overview from pilot work

Feedback was obtained from 14 service users and 16 clinicians, along with the diary review. Key findings were as follows:-

For staff, pre-pilot, there was anxiety about the additional pressure of implementing a new system, notwithstanding recognition of its theoretical merits. However, the CCaRM works best by recognising existing good practice and enhancing it. Thus, through the pilot experience, many recognised its advantages in streamlining care processes, supporting consistency of practice and better engaging service-users, commenting for example:

“(It is) *person centred and gives the individual a voice, focusses on what they find important and of value.”* (Nurse).

It offers:

“*…a good pictorial map*” (Nurse)

and it:

“…*structures information you gather about an individual*” (Nurse).

It was important for it to be activated as a multi-disciplinary project though, and not just for nurses. Where that was less successful, not everyone felt as engaged. Nevertheless, the approach was generally grasped well by staff, once experienced, but that needed consolidating through further experience and training. Staff were also interested in more use of CCaRM-based tools that might structure aspects of the care journey, for example routine case reviews.

For service-users, some preparation was possible for the CCaRM approach with good facilitation. However, it was in using the framework that it came to life for people. In this context there were noticeable shifts, to different degrees, from being focussed on wanting more staff input for themselves to:

(Wanting…) “…*more opportunities to prove myself”* (Service-User).

Further, care needs became more practically expressed, with a more developed view of the process as:

“*….people working together, making things better, and being involved*.” (Service-User).

The groundswell of interest and activation was recognised and reflected in the research diary, along with positive comments from a Care Quality Commission (CQC) inspection that took place. The research diary, in particular, captured the effort involved in preparing the ground for implementation, with a campaign of communication, workshops and coaching work. Although, this may have been effortful, the advantage was that it further stimulated a groundswell of interest that spilled over into other service areas that wanted to try the approach too. Of note was the enhanced sense of role and purpose that was witnessed amongst participants.

Some good examples of the practical benefits for particular cases were also captured by the research diary. Thus, one particular service-user was able to work through an easy read CCaRM ward round prompt sheet, ensuring their view was integrated into routine MDT discussions, despite lacking the confidence to go into meetings. Another service-user was previously troubled by multiple episodes of agitation with the need for segregation at times. Working with their key worker on “keeping people safe” within the CCaRM framework, they recovered function and remained incident free after 13 weeks. Similarly, a further four other individuals in the pilot were identified from routine reporting as being prone to multiple untoward incidents (i.e self-harm, need for restraint or seclusion etc.) Apart from one, where there were other compounding factors, all others recorded a fall to ‘no incidents’ over the course of the pilot.

Further reflection was found in the research diary to emphasise the importance of training the whole multi-disciplinary team (MDT) in the project, along with engaging good local leadership. In this context, two particular issues were faced. First, it was a challenge to dovetail the structure of the CCaRM process with the in-house electronic case record system. Developing CCaRM informed CPA case management review documentation would be a good example of this. Amending information systems and policies within organisations to support new frameworks in practice required engaging a fresh set of wider stakeholders within the organisation. Second, and related to this, wider competing organisational priorities are always a challenge. Therefore, maintaining high-level stakeholder engagement remained important.

In summary, this small-scale pilot found favour, particularly with early adopters, but attention to embedding in pre-existing IT and wider service systems is needed. It was experiencing the approach in action, using simple visual tools, that mattered. Adapting CCaRM based tools to the local context (such as templates for case reviews) particularly fostered multi-disciplinary engagement. Key themes for further implementation were the importance of an ongoing training plan, and an implementation team to plan, support and further facilitate its contribution to valued outcomes for all. On this basis, it was considered implementable by local managers. Further evaluation continues.

## Discussion

This paper presents the CCaRM approach at the “proof of concept stage”. It embraces a pragmatic agenda for local sense-making to drive “people powered” care [16], which would appeal to many involved in improving complex care practice. It offers a collaborative stance for coordinating and achieving “my best outcomes” [39]. It shows promise as a device for co-platforming value-based healthcare in a particularly complex area of practice.

Its limitations are that the empirical work so far is in the early stages, being relatively small scale and focussing on one specific service provider. Further evaluation would need to expand the mixed methods approach to involve more quantitative evaluation of service impact, and more in-depth evaluation of service user and carer experiences. Although, in principle, it ought to work in other areas of complex care practice, that would need further empirical exploration. Nevertheless, if the aim of integrated care is to reduce the experience of fragmentation of services [3,40], we would position the CCaRM approach as contributing coherence. First, it develops VBHC thinking as an integrating idea within case-level practice. Second, it contributes to positioning the case more assertively within wider care systems.

**Figure 2:**
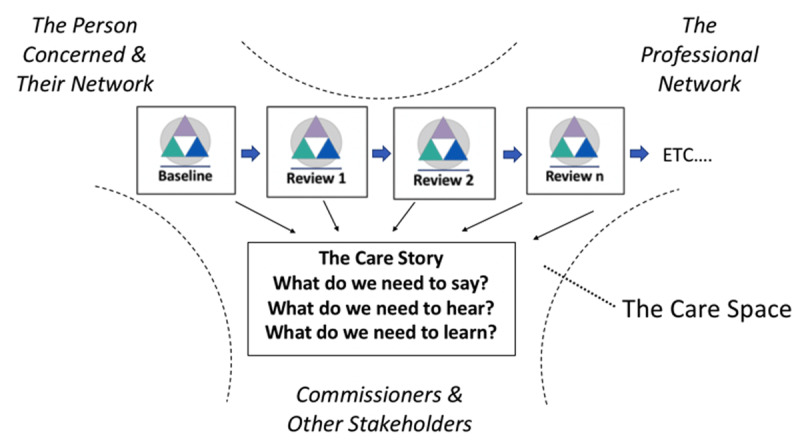
Co-platforming care over time, from case review to case review.

It is legitimate to draw conceptual inferences from case study examples to similar phenomena in other areas of complex healthcare [41]. On that basis, Figure 2 gives shape to how, in principle, a CCaRM based approach might impact on collaborative value generation in services more widely.

In this context, the first key feature is the focus on the case-based view as a coherent organisational form [28,33]. This aligns with forms of accountability envisaged in many case management approaches, as well as the CPA [7,30,31]. The more participants hold each other to account in case management, the stronger the care project becomes as a focus for VBHC. This platforming allows the case level project to become the primary focus of agency for “democratic” value generation.

However, equally, the case level care space is uniquely formed at the interface of wider participant networks [33,34]. In other words, participants (including commissioners, clinicians or family members) will also find themselves with a foot in other priority camps across the wider system, which may fluctuate over time. We noticed that tension in our pilot work in the playing out of the boundary between concern for the individual case, and a focus on more generic principles of the service pathway and wider care system perspectives. Thus, as positioned in Figure 2, how value-making is customised within cases, interacts with how the emergence of valued outcomes sits within participants’ wider networks of interests.

The second key feature is the structuring of the perspective of service-users and families into the care process, alongside other stakeholders. In this context, case level complexity is functionally, locally defined, however that arises, making sure nothing gets missed. The CCaRM can map issues relating to health conditions, to individuals, and to many other concerns (including local network and system constraints, prevailing service dynamics issues etc). In principle, it avoids service-users and families feeling they have to reach a threshold of “being complex” in order to gain a service: it is about how complexity is manifest in the individual case. Meanwhile, for service-user and carer experience, a golden thread of “what matters” connects, for example, conversations with key workers, care plan formation, MDT reviews and CPA reviews. Reflecting this, in our findings, participants welcomed a better streamlining and customising of care experience. Not only that, Figure 2 illustrates the individualised tone of reflective, “democratic” value-making that the CCaRM approach supports, aggregated from case review to case review. A welcome contribution to case management practice [42].

Finally, whilst more empirical work is needed to explore the impact and wider usefulness of the CCaRM approach, Figure 2 can be seen as itself a platform for collaborative research. Better understanding case-level value integration is of wider research interest [1,23]. The shift in perspective with the CCaRM approach is to make collaborative operationalisation the cornerstone of the care process, for example with the collaborative evaluation of complexity and valued outcomes as described. It also brings into focus the within-case and wider service system interplay. We would envisage further research inquiry as similarly involving collaborative methodologies. Meanwhile, an early question would be when and how such more intricate value-based co-platforming has advantages over simply improving the generalities of care, as for example the CCM is purported to do [5,8,24,25,43].

## Conclusion

The CCaRM is a practical, collaborative case-level service platform, with a clear focus on individualised value generation. It has made an important contribution to a local service, and, in principle, it could be adapted to other care settings. This is a novel contribution for integrating value-based healthcare into practice. It addresses a critical gap in support for service-users and practitioners in areas of complex practice. Its distinctiveness lies in the operationalisation of the value-based case view, the managing of complexity, the promotion of value-based case management, and development of co-platforming healthcare as a concept. There is more work to do to explore the transition to its service wide implementation, however this local experience encourages the view that this would be feasible. Experience suggests it needs to be seeded into services as a collaborative project, encouraging local ownership. Senior management support would be vital, with investment in a multi-disciplinary implementation team to support its roll-out and reconciliation with historic organisational practice. On that basis, there is encouragement to think that the CCaRM approach could potentially support case-level driven service transformation, and offers a useful foundation for further research.

## Appendix

**The CCaRM Process: Descriptions of example features of interest for each value theme** 

**Table:**

VALUE THEME	DESCRIPTION

1. Identifying the best possible circle of support	Map all the network of key people involved with the service-user in their care project. This draws on three sources: i. The service-user’s own network of family, friends and supporters. ii. The care coordinator and the network of clinicians involved. iii. Commissioners and other wider stakeholders as may be relevant. Explore what strengths and contributions each might bring, and has anyone been overlooked? What further development might be useful and how that can be achieved?

2. About Me: Developing a shared understanding	What is the care predicament, and to what extent is there a shared understanding? This might consider: i. Progress with engagement ii Progress with an agreed working theory or formulation iii Progress with an agreed process of further inquiry Explore where and how shared understanding is being built, and where and how it could be improved. Consider what useful tools or models might help deepen understanding.

3. My Problem Toolkits	How many distinct problem areas are there (mental or physical) that are having a key functional impact? How well are these areas supported? Consider: i What is the most useful way to all of presenting each problem area (for example as a diagnostic problem or a problem description). ii What is the right balance between capturing problem areas as interlinked, and capturing them as distinct? For each problem area, what is already in hand by way of assessing care planning and making progress? Consider what guidelines might be available. What further initiatives are needed to build a more rounded toolkit?

4. Joining In and Living a Good Life	What opportunities might there be for doing meaningful things, joining in activities and managing daily concerns? Consider: i. Communication: what works in supporting personal interactions (including verbal and non-verbal language, maturity and preferred style)? ii. The role of day-to-day structure (including daily routines, need for prompts and self-organisational skills) iii. Opportunities, personal interests, range of skills iv. What does it mean to “live a good life” [1]? Explore what progress has been made in exploring each of these areas. Consider what useful tools or models might help deepen understanding. Consider what further initiatives might help.

5. Keeping People Safe and Well	This involves the broad area of avoiding harm, either unintended or not. This includes thinking about risks arising from behaviour, and also issues of protection, promoting good health and supporting autonomy and personal reputation. Consider: i Risk behaviour (eg harm to self or others, deterioration in coping, vulnerability, offending concerns). ii Medication management needs iii Maintaining and promoting good physical and mental health iv Managing capacity, dignity and consent issues For each area, consider the progress made in developing collaborative assessments and care plans. Consider what further initiatives might be needed. Consider what tools, models, legal and rights frameworks might help.

6. What Progress Am I Making?	Considers how people will know that progress is being made as the care journey unfolds. There are three elements to this: i Agreement and review of relevant, available progress monitoring tools. ii Agreement and review of the case management process iii Democratic outcomes: That is, the quality of collaborative discussion, leading to a shared description and judgement. Also, definitive agreement on whether progress has been made or not against each value theme following a case management review [2]. Explore each element to identify what evaluation practices are already in place, and what further improvements could be made.

### Appendix References

1. Ward, T. (2002). The management of risk and the design of good lives. Australian Psychologist, 37(3), 172–179. https://doi.org/10.1080/00050060210001706846.
2. Spurrell M, Potts L, Shaw A. “The complex case and recovery management framework: The CCaRM.” Keynote presentation at the International Conference on the Care and Treatment of Offenders with an Intellectual Disability and/or Developmental Disability. 2019, 10–11 April, Birmingham, UK. https://www.autism.org.uk/what-we-do/professional-development/past-conferences/offenders-2019-presentations.
